# Higher monomeric C-reactive protein levels are associated with premature coronary artery disease

**DOI:** 10.3389/fimmu.2024.1501125

**Published:** 2025-01-10

**Authors:** Ivan Melnikov, Sergey Kozlov, Sergey Okhota, Olga Saburova, Yuliya Avtaeva, Tatiana Kuznetsova, Konstantin Guria, Lyudmila Prokofieva, Tatiana Riazantseva, Shang-Rong Ji, Yi Wu, Zufar Gabbasov

**Affiliations:** ^1^ Laboratory of Cell Hemostasis, Chazov National Medical Research Center of Cardiology of the Ministry of Health of the Russian Federation, Moscow, Russia; ^2^ Laboratory of Gas Exchange, Biomechanics and Barophysiology, State Scientific Center of the Russian Federation – The Institute of Biomedical Problems of the Russian Academy of Sciences, Moscow, Russia; ^3^ Department of Problems of Atherosclerosis, Chazov National Medical Research Center of Cardiology of the Ministry of Health of the Russian Federation, Moscow, Russia; ^4^ Laboratory of Neurohumoral Regulation of Cardiovascular Diseases, Chazov National Medical Research Center of Cardiology of the Ministry of Health of the Russian Federation, Moscow, Russia; ^5^ Laboratory of Human Stem Cells, Chazov National Medical Research Center of Cardiology of the Ministry of Health of the Russian Federation, Moscow, Russia; ^6^ Key Laboratory of Cell Activities and Stress Adaptations of Ministry of Education (MOE), School of Life Sciences, Lanzhou University, Lanzhou, China; ^7^ Key Laboratory of Environment and Genes Related to Diseases of Ministry of Education (MOE), School of Basic Medical Sciences, Xi’an Jiaotong University, Xi’an, Shaanxi, China

**Keywords:** C-reactive protein, monomeric C-reactive protein, mCRP, atherosclerosis, coronary artery disease, inflammation, residual inflammatory cardiovascular risk

## Abstract

**Introduction:**

Chronic inflammation is a major risk factor for coronary artery disease (CAD). Currently, the inflammatory cardiovascular risk is assessed via C-reactive protein (CRP) levels measured using a high-sensitivity assay (hsCRP). Monomeric CRP (mCRP) is a locally produced form of CRP that has emerged as a potential biomarker of inflammation.

**Aim:**

This study investigated whether mCRP levels are associated with premature CAD.

**Materials and methods:**

This study comprised 103 participants of both sexes, including 50 patients 56 ± 7 years old with premature CAD and 53 patients 51 ± 10 years old without CAD. CAD was verified using coronary angiography, hsCRP levels were measured using a standard assay, and mCRP levels were measured using fluorescent cytometric beads conjugated with an anti-mCRP antibody.

**Results:**

The levels of hsCRP were 0.99 (0.59; 3.10) mg/L vs. 0.63 (0.35; 1.85) mg/L (p = 0.067), and mCRP 6.84 (4.20; 13.78) µg/L vs. 2.57 (0.32; 5.66) µg/L (p <0.001) in patients with CAD vs. patients without CAD, respectively. There was a weak positive correlation between the mCRP and hsCRP levels (ρ = 0.214; p = 0.030). hsCRP levels were below 2.0 mg/L (i.e., residual inflammatory cardiovascular risk should have been excluded) in 70% of patients with CAD and 79% of patients without CAD (p = 0.365). mCRP levels differed between the groups of patients with hsCRP levels below 2.0 mg/L: 5.14 (4.07; 10.68) µg/L vs. 2.77 (0.53; 5.00) µg/L in patients with or without CAD, respectively (p <0.001). Logistic regression analysis demonstrated that mCRP levels were independently associated with premature CAD. The adjusted odds ratio was 1.18 (95% CI 1.06-1.33, p = 0.004) per each µg/L increase in mCRP levels.

**Conclusion:**

Higher mCRP levels were associated with premature CAD, independent of hsCRP levels and traditional risk factors.

## 1 Introduction

Chronic inflammation is a major risk factor for coronary artery disease (CAD) (1). C-reactive protein (CRP) is an acute-phase protein that is widely used as a reliable biomarker of inflammation (2). A level of CRP that is 2.0 mg/L or higher, as measured using a high-sensitivity assay (hsCRP), indicates the residual inflammatory cardiovascular risk (2).

CRP is produced by hepatocytes in a form of the pentameric disc (pCRP) that consists of five subunits bound by disulfide bonds (3). Each subunit on one side of this disc presents binding sites for lysophosphocholine and calcium ions, as well as cholesterol. The opposite side of the pCRP disc presents binding sites for C1q complement and Fc gamma receptors (4). As a component of the innate immune system, pCRP functions as a pattern recognition molecule and regulates host defense responses (3). It has been suggested that pCRP exerts weak anti- and proinflammatory actions (5).

The profile of the biological actions of CRP dramatically changes when pCRP binds to lysophosphocholine on the membranes of damaged cells and their microparticles (3, 6). There, the disulfide bonds between pCRP subunits undergo reduction. This results in the exposure of a C-terminal neoepitope (a.a. 199–206) that possesses antigenicity differing from that of the intact pCRP disk. The initial conformational change is the formation of pCRP*, which retains overall pentameric symmetry, while expressing the neoepitope. Braig et al. demonstrated that pCRP* is a major tissue-bound form of CRP, which is able to activate the complement system (7). Further conformational rearrangement leads to the complete dissociation of the pCRP disc into separate monomeric subunits (mCRP). mCRP exerts potent proinflammatory actions (3, 4), can induce endothelial activation (8) and can promote the recruitment of leukocytes (9), T-lymphocyte extravasation (10) and thrombus growth (11–13). *In vivo* evidence suggests that mCRP can stimulate the infiltration of monocytes into myocardial (6, 14) and renal tissues (15) after ischemia/reperfusion injury. The biologic actions of mCRP are discussed in more detail elsewhere (3, 4, 16).

Conventional hsCRP assays primarily detect pCRP in blood plasma or serum. The measurement of mCRP levels requires aptamers or antibodies that recognize the neoepitope of the monomeric subunits and do not recognize pCRP. In recent years, the first reports measuring the levels of mCRP in humans have been published (17). This study aimed to investigate whether mCRP levels are associated with premature CAD.

## 2 Materials and methods

### 2.1 Study design

Premature CAD was defined as the development of CAD in males before 55 years of age or females before 65 years of age (18).

This was a case–control study. Study participants were enrolled from patients admitted to Chazov National Medical Research Center of Cardiology, Moscow, Russia. Males under 55 years of age with the onset of CAD before 50 years of age and females under 65 years of age with the onset of CAD before 60 years of age were eligible for inclusion in the CAD group. Patients without clinical manifestations of CAD and stenotic atherosclerotic lesions in the coronary arteries were eligible for inclusion in the control group.

Prior to enrollment, all patients were assessed for clinical manifestations of CAD and coronary angiography. Atherosclerotic lesions were considered stenotic if they narrowed 50% or more of the lumen of the major coronary arteries (the left main, left anterior descending, left circumflex, and right coronary arteries or their branches with a diameter larger than 2 mm) (19).

The exclusion criteria were familial hypercholesterolemia, low-density lipoprotein cholesterol (LDL-C) levels higher than 4.9 mmol/L, congenital or acquired coagulation disorders, unstable angina, a period within the first 2 months after myocardial infarction, heart failure NYHA class III–IV, a left ventricle ejection fraction less than 40%, the permanent form of atrial fibrillation, aortic or mitral stenosis, malignant tumors, or an episode of acute inflammation within 2 months of the study.

Patients meeting the inclusion criteria were asked if they would like to participate in the study. The study included 50 patients (37 male and 13 female) with stable CAD and 53 patients (22 male and 31 female) without CAD.

### 2.2 mCRP measurement

This assay utilized cytometric beads (Becton-Dickinson, USA, catalog #560031) conjugated with an anti-mCRP monoclonal antibody clone CRP-8 (Sigma-Aldrich, USA, catalog #C1688). This antibody is specific for mCRP (20–22). Bead-to-antibody binding was performed using Sulfo-SMCC (Sigma-Aldrich, USA, catalog #M6035) and dithiothreitol (Thermo Fisher, USA, catalog #R0861) according to the manufacturer’s protocol. A FITC-labelled polyclonal anti-CRP antibody was used as a detector antibody (ImTek, Russia, catalog #GAHCrp). Recombinant mCRP (a gift by Dr. Yi Wu, Xi’an Jiaotong University, China) was used for calibration (21, 23). Prior to flow cytometry, the samples were incubated with CRP-8-conjugated beads in a dark place at room temperature for 60 min. All measurements were performed using the FACS Canto II flow cytometer (Becton-Dickinson, USA). The assay is described in detail elsewhere (20).

### 2.3 Blood sample collection and analysis

Blood samples from the cubital vein were collected into S-Monovette 3.8% sodium citrate vials (Sarstedt, Germany) after 12 hours of fasting. Platelet-poor plasma was prepared via centrifugation at 2000 g for 20 min and stored at -70°C. Prior to measurement, the samples were thawed in a water bath at 37°C. The levels of hsCRP were measured using latex-enhanced immunonephelometry on a BN ProSpec analyzer (Dade-Behring, Germany) using the CardioPhase hsCRP assay (Siemens, Germany, catalog #10446090).

### 2.4 Statistical analysis

The data are presented as mean ± standard deviation or median (lower quartile; upper quartile) values, as appropriate. The distribution was determined using the Shapiro-Wilk W test. Correlations were assessed using Spearman’s rank correlation coefficient (ρ). A comparative analysis of the two independent groups was performed using the Mann-Whitney U test for quantitative data and Fisher’s exact test for qualitative data. Logistic regression analysis was used to detect the independent risk factors associated with an increased likelihood of CAD. The variables included in the regression model were selected using the Wald test. The quality of the model was assessed using the Hosmer-Lemeshow goodness-of-fit test, the Nagelkerke R^2^ test and ROC analysis. The results of ROC analysis and the Youden test were used to calculate the optimal cut-off value. Statistical significance was set at p <0.05. All statistical tests were two-tailed. The study was considered sufficiently powered to evaluate a difference between groups of patients at α = 0.90. Statistical analysis was performed using IBM SPSS Statistics (version 26.0; SPSS Inc., USA).

### 2.5 Ethical approval

The study followed Good Clinical Practice standards and the principles of the Declaration of Helsinki. This study was approved by the ethics committee of the Chazov National Medical Research Center of Cardiology of the Ministry of Health of the Russian Federation, Moscow, Russia. Written informed consent was obtained from all participants.

## 3 Results

The clinical characteristics of patients with and without CAD are provided in **Table 1**. Patients with CAD were older and more likely to be male. Furthermore, patients with CAD had a higher prevalence of hyperlipidemia, smoking, and carotid atherosclerosis than patients without CAD. Patients with CAD had lower levels of total cholesterol and LDL-C due to the lipid-lowering therapy.

**Table 1:** Clinical and laboratory characteristics of patients.

	With CAD (n = 50)	Without CAD (n = 53)	p
Age, years	56 ± 7	51 ± 10	0.006
Male/female, no. (%)	37 (74%)/13 (26%)	22 (42%)/31 (58%)	0.001
Family history of CAD, no. (%)	13 (26%)	8 (15%)	0.223
Hyperlipidemia, no. (%)	48 (96%)	37 (70%)	<0.001
Smoking, no. (%)	34 (68%)	18 (34%)	0.001
Obesity, no. (%)	33 (66%)	35 (66%)	0.998
Diabetes mellitus, no. (%)	12 (24%)	5 (9%)	0.063
Arterial hypertension, no. (%)	44 (88%)	42 (79%)	0.292
Carotid atherosclerosis, no. (%)	45 (90%)	35 (66%)	0.001
Total cholesterol, mmol/L	4.01 (3.24; 4.75)	5.38 (4.60; 6.42)	<0.001
LDL-C, mmol/L	1.88 (1.51; 2.68)	3.21 (2.53; 4.01)	<0.001
HDL-C, mmol/L	1.02 (0.88; 1.15)	1.25 (1.05; 1.48)	<0.001
Triglycerides, mmol/L	1.95 (1.39; 2.69)	1.64 (1.14; 2.16)	0.072

CAD, coronary artery disease; LDL-C, low-density lipoprotein cholesterol; HDL-C, high-density lipoprotein cholesterol.

The onset of CAD occurred at a mean age of 51 ± 6 years. In 48% of cases, the onset of CAD was caused by acute myocardial infarction. In total, 14% of patients with CAD had a history of recurrent acute cardiovascular events; 76% had a history of coronary artery stenting; and 10% had a history of coronary artery bypass grafting. Furthermore, 64% of patients had a history of repeat revascularization. Multivessel CAD was present in 66% of patients.

In total, 62% of patients with CAD received statin monotherapy; 32% received statins in combination with other lipid-lowering agents; 32% received aspirin monotherapy, 10% received P2Y12 inhibitor monotherapy; and 52% received dual anti-platelet therapy. Furthermore, 30% of patients without CAD received lipid-lowering therapy with statins only or in combination with other agents, and 23% received aspirin monotherapy.

**Figure 1:**
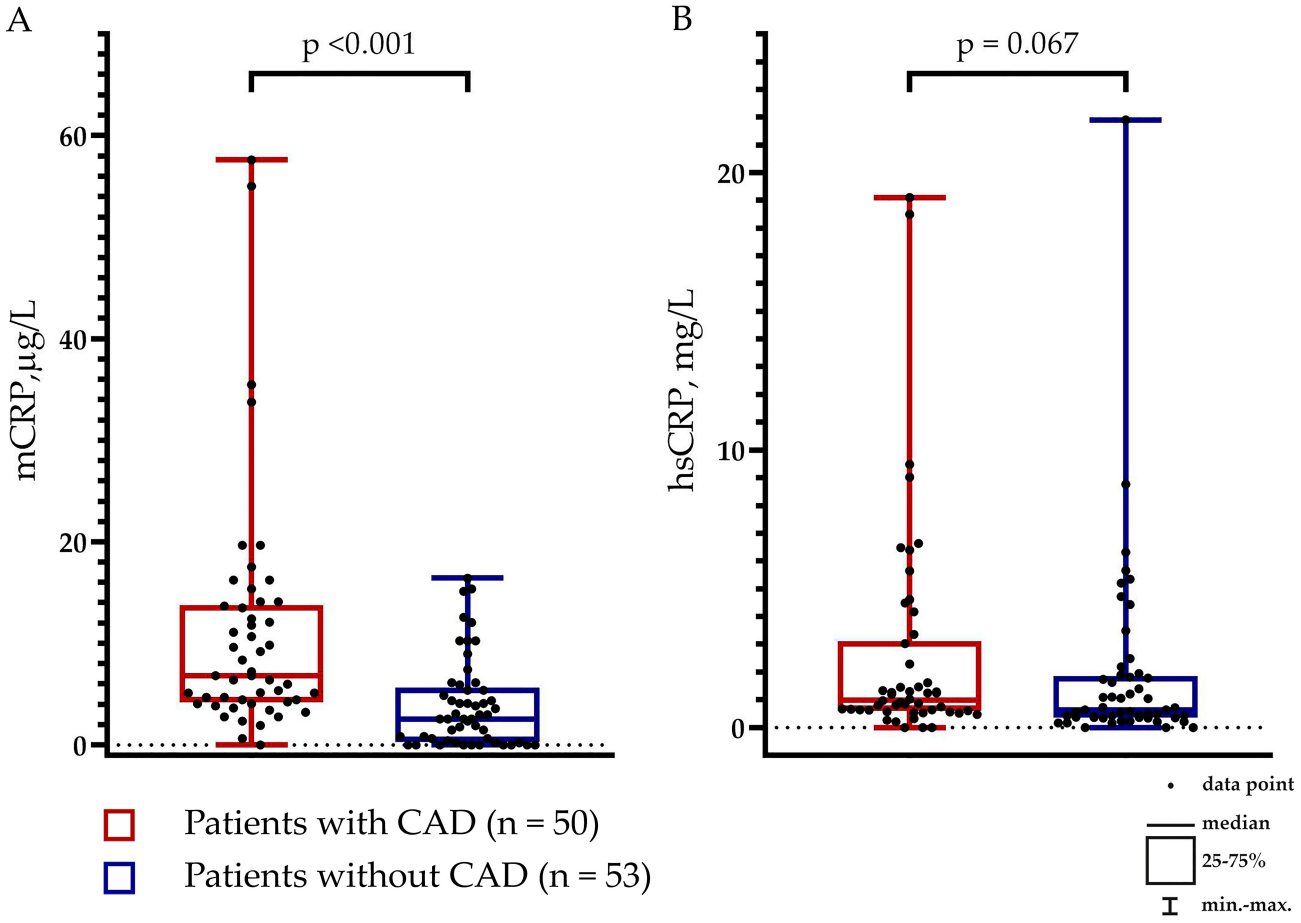
Levels of **(A)** Monomeric C-reactive protein (mCRP) and **(B)** pentameric C-reactive protein (pCRP) measured using high-sensitivity assay (hsCRP) in patients with (n = 50) and without (n = 53) coronary artery disease (CAD).

**Figure 2:**
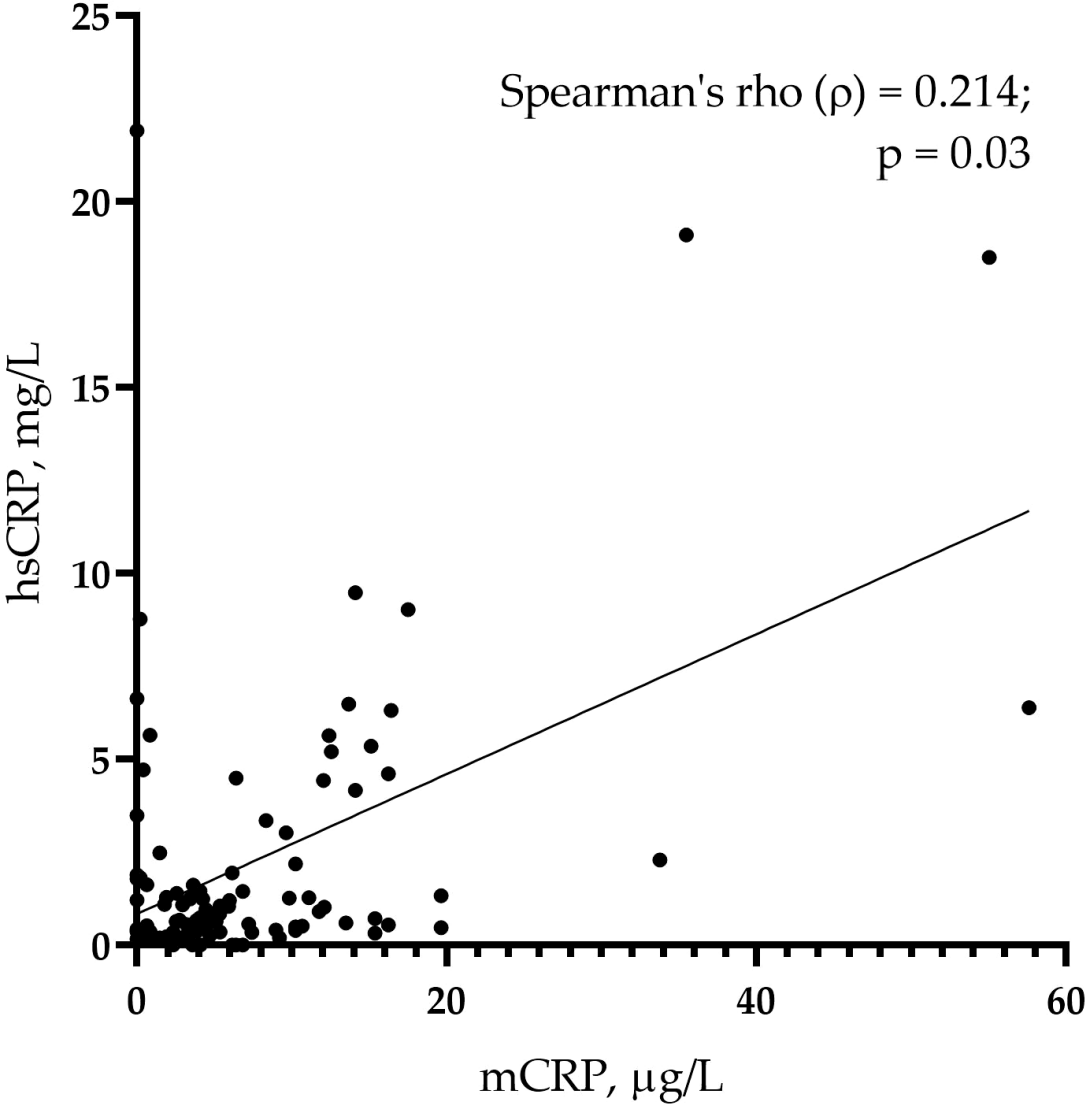
A correlation of monomeric C-reactive protein (mCRP) and pentameric C-reactive protein (pCRP) measured using high-sensitivity assay (hsCRP) in the whole sample (n = 103) of study participants.

The levels of mCRP were higher in patients with than without CAD: 6.84 (4.20; 13.78) µg/L vs. 2.57 (0.32; 5.66) µg/L, respectively (p <0.001) (**Figure 1A**). The levels of hsCRP did not differ between the groups: 0.99 (0.59; 3.10) mg/L vs. 0.63 (0.35; 1.85) mg/L in patients with vs. without CAD, respectively (p = 0.067) (**Figure 1B**). The mCRP/hsCRP ratio was 5.13 (2.77; 11.86) vs. 2.94 (0.11; 7.92) in patients with vs. without CAD, respectively (p = 0.022). There was a weak positive correlation between the levels of mCRP and hsCRP (ρ = 0.214; p = 0.030) (**Figure 2**). The study was adequately powered (0.98) to evaluate the difference in mCRP levels between the groups of patients.

**Figure 3:**
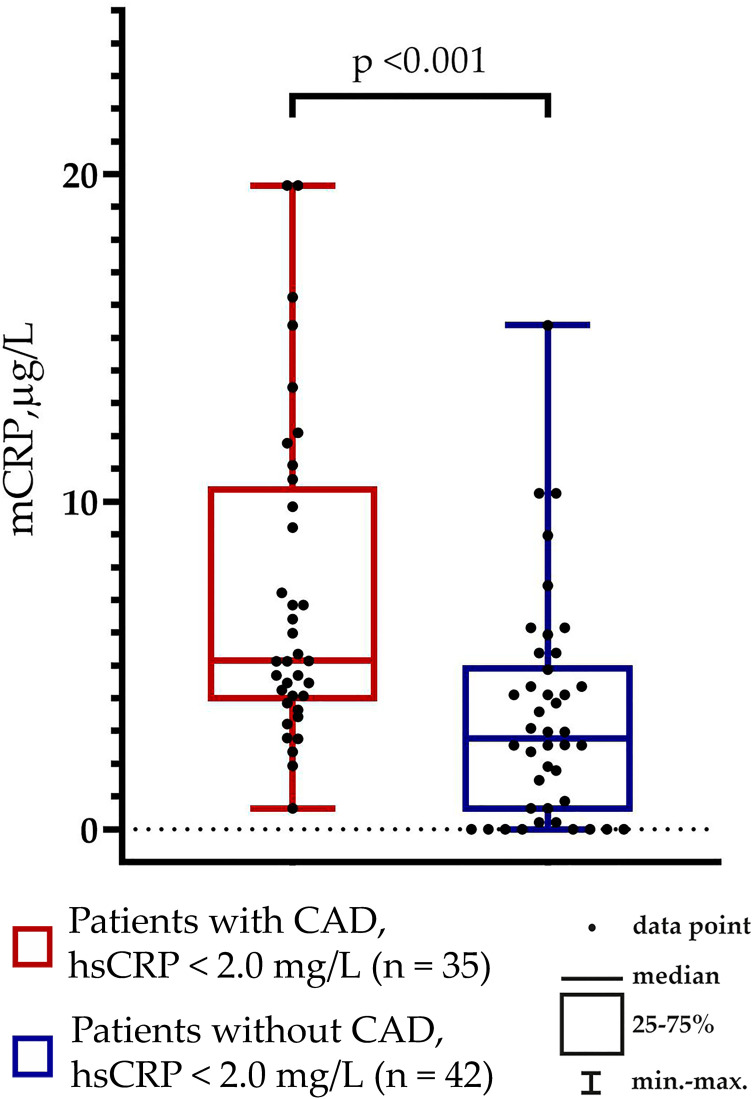
Monomeric C-reactive protein (mCRP) levels in patients without residual inflammatory cardiovascular risk (hsCRP levels below 2.0 mg/L).

hsCRP levels were below 2.0 mg/L (i.e., residual inflammatory cardiovascular risk should have been excluded) in 70% of patients with CAD and 79% of patients without CAD (p = 0.365). Notably, the mCRP levels differed between the groups of patients with hsCRP levels below 2.0 mg/L: 5.14 (4.07; 10.68) µg/L vs. 2.77 (0.53; 5.00) µg/L in patients with vs. without CAD, respectively (p <0.001) (**Figure 3**).

Associations of mCRP levels with traditional risk factors and hsCRP levels were analyzed using a correlation matrix and the logistic regression analysis. This was done in three steps. First, using a correlation matrix we found that there were no correlations between traditional risk factors for CAD and mCRP levels, and a weak positive correlation between mCRP and hsCRP levels. These factors were then analyzed using the univariate logistic regression analysis (**Table 2**). Then, according to the Wald criterion (p <0.05), the factors were included into the multivariate logistic regression model (**Table 3**). This model allowed adjustment of mCRP level odds ratio to odds ratios of other risk factors. The model with the highest percent of correct predictions (81.6%) included age, male sex, smoking and mCRP levels (**Table 3**). The Hosmer–Lemeshow test for the model was 0.606, which shows that the observed event rate matched the event rate expected in the subgroups of the model population. The Nagelkerke R^2^ was 0.537, that is, the model was acceptable for use. The adjusted odds ratio for premature CAD was 1.18 (95% CI 1.06-1.33, p = 0.004) per each µg/L increase in the mCRP levels.

**Table 2:** Univariate logistic regression analysis of the associations between independent factors and premature coronary artery disease.

Variable	Coefficient (β)	Standard error	Wald	p	Odds ratio (95% confidence interval)
mCRP, µg/L	0.175	0.05	13.41	<0.001	1.19 (1.09-1.31)
Smoking	1.42	0.42	11.43	0.001	4.13 (1.82-9.40)
Male sex	1.39	0.43	10.62	0.001	4.01 (1.74-9.25)
Hyperlipidemia	2.34	0.78	8.97	0.003	10.38 (2.25-47.99)
Age, years	0.074	0.03	8.14	0.004	1.08 (1.02-1.13)
Diabetes mellitus	1.11	0.58	3.72	0.054	3.03 (0.98-9.35)
Family history of CAD	0.68	0.50	1.85	0.174	1.98 (0.74-5.28)
Arterial hypertension	0.65	0.55	1.40	0.237	1.92 (0.65-5.66)
hsCRP, mg/L	0.06	0.06	1.01	0.315	1.06 (0.95-1.19)
Obesity	-0.001	0.24	<0.001	0.998	1.0 (0.62-1.61)

mCRP, monomeric C-reactive protein; CAD, coronary artery disease. Factors range from the lowest to the highest p value.

**Table 3:** Multivariate logistic regression analysis of the associations between independent factors and premature coronary artery disease.

Variable	Coefficient (β)	Standard error	Wald	p	Odds ratio (95% confidence interval)
mCRP, µg/L	0.17	0.06	8.26	0.004	1.18 (1.06-1.33)
Age, years	0.12	0.04	8.24	0.004	1.13 (1.04-1.22)
Male sex	2.33	0.68	11.76	0.001	10.33 (2.71-38.94)
Smoking	1.33	0.55	5.84	0.016	3.78 (1.27-11.12)
Intercept	-9.68	2.62	13.68	<0.001	–

mCRP, monomeric C-reactive protein.

**Figure 4:**
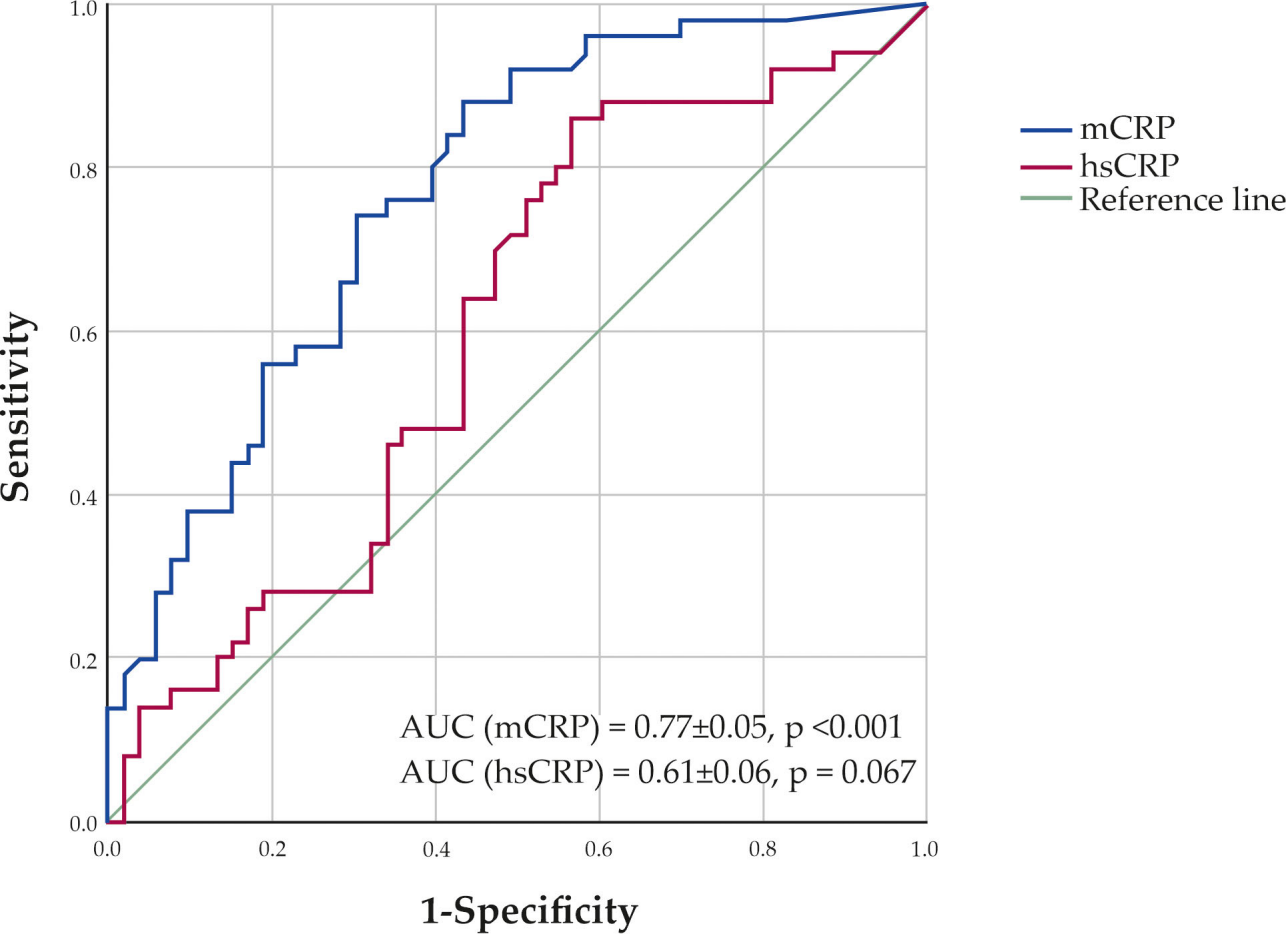
ROC curves showing sensitivity and specificity of monomeric C-reactive protein (mCRP) and high-sensitivity C-reactive protein (hsCRP) levels in classification of patients to the CAD group.

According to the ROC analysis, the optimal cut-off level for mCRP was 4.41 µg/L. At this level, patients were correctly classified into the CAD group with 74.0% sensitivity and 69.8% specificity (**Figure 4**). The area under the curve (AUC) was 0.77 ± 0.05 (95% CI 0.68-0.86; p <0.001). The AUC for hsCRP was 0.61 ± 0.06 (95% CI 0.49-0.72; p = 0.067).

As the ratio of males and females between patients with and without CAD was different, we analyzed whether this could cause any bias in the association between mCRP levels and premature CAD. The levels of mCRP did not differ between males and females: p = 0.772 for the whole sample of 103 patients (59 males vs. 44 females); p = 0.141 for the group of patients with CAD (37 males vs. 13 females); and p = 0.094 for the group of patients without CAD (22 males vs. 31 females). Furthermore, the ROC analysis classified patients into the CAD group with an AUC of 0.816 ± 0.057 (95% CI 0.705-0.928, p <0.001) for males and 0.814 ± 0.066 (95% CI 0.685-0.942, p = 0.001) for females. The optimal cut-off levels for the mCRP levels were 4.41 µg/L (70.3% sensitivity and 81.8% specificity) for males and 4.17 µg/L (92.3% sensitivity and 61.3% specificity) for females. Therefore, we concluded that the bias was unlikely.

## 4 Discussion

This study demonstrated that mCRP levels were higher in patients with CAD than in controls without CAD. Higher mCRP levels were associated with premature CAD, with an adjusted odds ratio of 1.18 (95% CI 1.06-1.33) per each µg/L increase in the levels of mCRP. This association was independent of traditional risk factors and hsCRP levels. Furthermore, in the study participants who presumably had no residual inflammatory risk (that is, hsCRP levels below 2.0 mg/L), the levels of mCRP were still higher in patients with CAD than without CAD.

To date, mCRP levels have been studied in humans with regard to a number of pathologies, including atherosclerotic cardiovascular disease (20, 24), osteoarthritis (25), acute inflammation (26), chronic obstructive pulmonary disease (27), autoimmune (28–30) and skin disorders (21), periodontitis (31), hepatitis B cirrhosis (32) and COVID-19 (33, 34). To our knowledge, few studies have reported the measurement of mCRP levels in atherosclerotic cardiovascular disease. Wang et al. demonstrated that mCRP levels were elevated in patients with acute myocardial infarction, whereas in patients with angina pectoris and healthy volunteers mCRP levels were below the detection limit. It is noteworthy that among patients with myocardial infarction, mCRP levels were higher in those with a poor outcome (24). Furthermore, a study by Wu et al. demonstrated that among patients with antineutrophil cytoplasmic antibody-associated vasculitis, mCRP levels were higher in patients with acute myocardial infarction than in those without it (30). In a recent study, we measured mCRP levels in individuals with asymptomatic carotid atherosclerosis and an initially moderate cardiovascular risk, performing follow-up for seven years. Higher mCRP levels were independently associated with an increase in the number and height of carotid atherosclerotic plaques by the end of the follow-up period. Noteworthy, this association was observed in individuals with normal hsCRP levels (20).

The mCRP to hsCRP ratio differed between patients with and without CAD, although the association of this ratio with CAD was weaker than that of mCRP alone. Some studies previously demonstrated that the ratio of monomeric to pentameric forms of CRP was substantially elevated in some inflammatory autoimmune diseases, particularly adult-onset Still’s disease (29) and age-related macular degeneration (35). Another study demonstrated that a higher ratio of monomeric to pentameric CRP enabled researchers to differentiate between active and quiescent disease states in patients with systemic lupus erythematosus (28).

Furthermore, the mCRP and hsCRP levels demonstrated a weak positive correlation in the current study. Some studies previously demonstrated a positive or negative correlation between the mCRP and hsCRP levels. On the contrary, a number of studies reported the absence of a correlation between these two forms of CRP (17). hsCRP primarily detects pCRP, which is produced in hepatocytes and secreted into the bloodstream (3). The production of pCRP is regulated by proinflammatory cytokines, mainly interleukin-6. Interleukin-6 is a link of the central inflammatory cascade (2). This cascade leads to the transduction of inflammatory signaling from the NLRP3-inflammasome in damaged tissues through interleukin-1β and interleukin-6 to their ligand receptors (2, 36). NLRP3-inflammasome is activated under stimuli from endogenous damage-associated patterns (DAMPs), which include cholesterol crystals (37) and oxidized LDL (38, 39). The inflammasome converts pro-interleukin-1β into its active form, interleukin-1β, which is released from a number of cells to transcend complex signaling (36, 40). In particular, interleukin-1β stimulates the cellular release of interleukin-6. In its turn, interleukin-6 further transcends a wide range of stimuli, including the upregulation of pCRP production in hepatocytes (41). pCRP is considered the end product of the central inflammatory cascade (2). As such, pCRP is a biomarker that reflects the overall activity of the upstream links of this cascade (2, 36). mCRP is produced otherwise. One known pathway is the dissociation of pCRP (6, 42, 43). pCRP opsonizes the membranes of damaged and activated cells and their microparticles. There, pCRP undergoes dissociation into monomeric subunits (44). Another possible pathway of mCRP production is local synthesis. Macrophages and adipocytes have been shown to produce mCRP (45–48). Moreover, unlike pCRP, mCRP has been detected in damaged tissues in a number of histological studies (17, 43, 49). Therefore, the pathways of pCRP and mCRP production are at least partially different. This makes the relationship between mCRP and hsCRP levels non-linear, with alterations in different diseases and subsets of patients.

This study comprised patients with premature CAD, which develops in younger individuals. This is an aggressive form of CAD that is associated with frequent acute cardiovascular events and high death rates, which cannot be restrained even by the rigorous control of traditional risk factors (50). In addition, recent clinical trials have demonstrated the remarkable effectiveness of anti-inflammatory agents in the prevention of cardiovascular events, with colchicine being introduced into cardiovascular prevention guidelines (1, 51). The assessment of residual inflammatory cardiovascular risk can confer a substantial benefit in this context. Currently, this risk is assessed using hsCRP levels, which reflect the activity of the central inflammatory cascade. The complementary evaluation of the local inflammatory response using mCRP levels could possibly aid in the assessment of residual inflammatory cardiovascular risk.

## 5 Conclusion

This study revealed that mCRP levels exhibit an independent association with premature CAD, which was more pronounced than the association of hsCRP levels with premature CAD. Moreover, this association was present even in patients with hsCRP levels below 2.0 mg/L, in whom residual inflammatory risk should have been excluded.

## Data Availability

The raw data supporting the conclusions of this article will be made available by the authors, without undue reservation.
